# Extracellular Vesicle-Associated RNA as a Carrier of Epigenetic Information

**DOI:** 10.3390/genes8100240

**Published:** 2017-09-22

**Authors:** Carlo Maria Di Liegro, Gabriella Schiera, Italia Di Liegro

**Affiliations:** 1 Department of Biological Chemical and Pharmaceutical Sciences and Technologies (STEBICEF), University of Palermo (UNIPA), I-90128 Palermo, Italy; carlomaria.diliegro@unipa.it (C.M.D.L.); gabriella.schiera@unipa.it (G.S.); 2 Department of Experimental Biomedicine and Clinical Neurosciences (BIONEC), University of Palermo, I-90127 Palermo, Italy

**Keywords:** mRNA, non-coding RNA (ncRNA), RNA-binding proteins (RBPs), extracellular vesicles (EVs)

## Abstract

Post-transcriptional regulation of messenger RNA (mRNA) metabolism and subcellular localization is of the utmost importance both during development and in cell differentiation. Besides carrying genetic information, mRNAs contain cis-acting signals (zip codes), usually present in their 5′- and 3′-untranslated regions (UTRs). By binding to these signals, trans-acting factors, such as RNA-binding proteins (RBPs), and/or non-coding RNAs (ncRNAs), control mRNA localization, translation and stability. RBPs can also form complexes with non-coding RNAs of different sizes. The release of extracellular vesicles (EVs) is a conserved process that allows both normal and cancer cells to horizontally transfer molecules, and hence properties, to neighboring cells. By interacting with proteins that are specifically sorted to EVs, mRNAs as well as ncRNAs can be transferred from cell to cell. In this review, we discuss the mechanisms underlying the sorting to EVs of different classes of molecules, as well as the role of extracellular RNAs and the associated proteins in altering gene expression in the recipient cells. Importantly, if, on the one hand, RBPs play a critical role in transferring RNAs through EVs, RNA itself could, on the other hand, function as a carrier to transfer proteins (i.e., chromatin modifiers, and transcription factors) that, once transferred, can alter the cell’s epigenome.

## 1. Introduction

The release of extracellular vesicles (EVs) by a producing cell and their fusion with surrounding, recipient cells is probably an ancient process. It is, indeed, highly conserved in evolution from bacteria [1,2] to human cells [3], and, most importantly, there is evidence of inter-specific transfer of EVs, even from microorganisms to mammal cells [4]. As we will discuss, EVs contain a collection of specifically sorted proteins, RNAs and lipids, thus representing an astonishing tool for horizontally transferring biochemical properties from cell to cell, and perhaps for leveling out biological potentials in a population.

Central protagonists of EV-mediated exchanges are RNAs or, more precisely, RNA–protein complexes. Similar to viruses that bud from infected cells, ready to infect other cells, extracellular RNA–protein complexes can reach cells other than the producers and, once inside, can modify gene expression in many ways. In the last decade, much attention has focused on the RNA component of the complexes. Thus, we now know that EVs can transfer various classes of RNAs, from messenger RNAs (mRNAs), encoding for specific proteins [5], to non-coding RNAs (ncRNAs) of different sizes, among which are long non-coding RNAs (lncRNAs) [6], microRNAs (miRNAs) [5], and circular RNAs (circRNAs) [7]. mRNAs may be translated in the recipient cells, thus providing them with new proteins [8,9]. miRNAs may target the mRNAs of the recipient cells, decreasing their translation and/or increasing their degradation, thus depleting cells of specific resident proteins [10]. Finally, circRNAs seem to function in many cases as sponges, able to bind miRNAs and decrease their mRNA-targeting capacity [11]. In general terms, lncRNAs seem to serve as decoy factors, able to trap, for example, transcription factors and/or other regulatory proteins, thus impeding their interaction with chromatin in the recipient cells. lncRNAs have also been suggested to act as scaffolds for formation and/or localization of protein complexes that regulate gene expression at the transcriptional and/or post-transcriptional level [12]. Interestingly, it is now emerging that mRNAs (and in particular their 3′-untranslated regions, 3′-UTR) may share some functions with non-coding RNAs in that they can bind and trap proteins [13]. Thus, coming back to EVs, both coding and non-coding RNAs in vesicles could act as protein carriers that transport proteins from one cell to another; once in the recipient cells, these proteins may bind and modify chromatin structure and activity, acting as epigenetic players able to modify gene expression.

In this review, we will discuss these hypotheses in the light of the transformative activity that EVs seem to have in cancer [14], and of the apparent ‘infectious’ potential attributed to them in some neurodegenerative diseases [15].

## 2. Role of RNA-Binding Proteins in RNA Metabolism and Localization

In eukaryotes, gene expression is realized by a series of sequential events during which mRNA molecules are produced, processed, transported to the cytoplasm, and translated into proteins. The control of the whole process is ensured by a cohort of proteins, directly or indirectly interacting with mRNAs and with each other, which also determine mRNA stability, localization, and translational levels. In other words, mRNA is not a naked molecule wandering in the cell, but a component of changing complexes, which include RNA binding proteins (RBPs) and other regulatory RNAs. It is generally thought that, during the first steps of the mRNA life, a core group of ”general” proteins is put in place, guided by common features such as the 5′-cap, splicing sites, and poly(A)-tail; thereafter, more “specific” proteins should be positioned on RNA, determining its frequency of transport to the cytoplasm, its trafficking to the final destination, and, finally, its translational level. This view is, however, an oversimplification, because RBPs display a wide range of binding specificity [16]. In addition, each mRNA interacts with many different RBPs, and each RBP can interact with hundreds of mRNAs. In the end, each mRNA is complexed with a unique set of regulatory molecules, which, together with the mechanisms and rules of interpretation of the whole system, constitutes the so-called mRNP code [17].

The number of identified RBPs has rapidly increased in the last 10 years and the family now includes more than 1000 members. By recognizing and binding specific sequences or stem-loop structures, often located in the 3′-UTR, RBPs control all steps of the mRNA metabolism, from transcription to translation [18,19,20,21].

RBPs also interact with non-coding RNAs, and it is likely that competition for RBP binding among RNA molecules of different classes has a role in controlling gene expression; moreover, at least in some cases, ncRNAs and mRNAs might be part of ternary ncRNA–mRNA–protein complexes. In addition, RBPs can participate in the maturation of miRNAs [22], and in the formation of some circRNAs [23] (see Section 5).

Actually, RBP functions are even more complex than thought before. For example, they are probably even involved in maintenance of genome integrity [24], and overexpression in yeast of YRA1, a member of the family of the heterogeneous nuclear ribonucleoprotein hnRNP-like export factors, has been shown to cause replication impairment, DNA damage, and telomere shortening [25].

In general terms, RBPs are very often multifunctional proteins, acting at different stages of gene expression. For example, Wilms’ tumor 1 (WT1) factor has been described as a transcriptional regulator [26], but it has been recently demonstrated to also be an RBP that binds to secondary structures in the 3′-UTR of developmentally regulated mRNAs, thus influencing their stability [27].

One category of RBPs that is attracting much interest is represented by enzymes already known for their involvement in biochemical pathways, and for which participation in some step of the RNA metabolism has been more recently demonstrated [28]. For example, glyceraldehyde-3-phosphate dehydrogenase (GAPDH), a glycolytic enzyme, binds to the 3′-UTR of the interferon-γ mRNA and represses its translation in inactive T-lymphocytes. When glycolysis is enhanced in activated T-lymphocytes, and GAPDH works as an aerobic enzyme, the translation of interferon-γ mRNA is no longer repressed [29]. Similarly, superoxide dismutase 1 (SOD1) also functions as an RNA binding protein [30].

Among RBP’s multiple functions, one that is of particular interest is their involvement in RNA localization. In the past, RNA localization was considered an unusual mechanism, operating in a few specific cell types, such as developing embryos and polarized cells (e.g., neurons), while, nowadays, an increasing number of studies indicate that it is a quite common, evolutionarily conserved process [31,32]. The understanding of the whole trafficking process, however, requires the identification of the RBP domains and nucleic acid sequences/structures (zip codes) involved [33], as well as of the mechanisms allowing the movements of RNA–protein complexes inside the cells, most probably driven by specific motor proteins [34,35].

## 3. Extracellular Vesicles as Vehicles for the Horizontal Transfer of Molecules

The term ‘exosome’ was first used to describe vesicles expulsed by exocytosis from a multivesicular body (MVB), during reticulocyte maturation into erythrocytes [36]. These first studies also demonstrated that in this way maturing reticulocytes discarded unwanted or obsolete molecules, such as transferrin receptors [37,38].

Extracellular vesicles have been now recognized as a universal intercellular communication route, both in prokaryotes and eukaryotes. Thanks to their ability to transfer proteins, lipids and nucleic acids, EVs can affect cell functions in both physiological and pathological conditions [39]. They have also been found in biological fluids such as serum, saliva, amniotic fluid and synovial fluid, breast milk, and urine [40].

EVs are particles of spheroidal morphology, surrounded by a membrane consisting of a lipid bilayer, in which proteins are also present, and differ in size, biogenesis, and release mechanisms [41]. EVs can be classified into at least two large classes: (i) microvesicles (MVs), with dimensions in the range of 100 nm–1 μm, that are directly shed from the plasma membrane through a process resembling virus budding, and (ii) exosomes, which are smaller vesicles of 50–100 nm in diameter, generated by exocytosis of multivesicular bodies. In addition, apoptotic bodies also constitute a significant proportion of vesicles derived from cells and circulating in the body [42].

The term ‘extracellular vesicles’ has often been used in the scientific literature in a imprecise way [43]. During the meeting held in Washington, D.C. in 2015, the International Society for Extracellular Vesicles (ISEV) also evidenced the heterogeneous nature of EVs and highlighted that: (i) EVs indistinguishable from exosomes are directly released from the plasma membrane; (ii) diameters up to 250 nm have been reported for exosomes; and (iii) proteins such as tetraspanins, sometimes considered characteristic components of exosomes, are not unique to them. However, until now the exact definitions have still varied widely among publications. In an attempt to standardize the somehow confusing nomenclature, the last ISEV position paper proposed a classification based on the experimental procedures used to purify EVs: (i) vesicles sedimenting at 100,000× *g* as “small EVs” (sEVs) rather than exosomes, (ii) those pelleting at an intermediate speed (lower than 20,000× *g*) as “medium EVs” (mEVs, including microvesicles or ectosomes), and (iii) those pelleting at low speed (e.g., 2000× *g* as “large EVs” (lEVs, including large fragments of the releasing cell and large apoptotic bodies) [3].

Obviously, if found, specific EV components could identify different EV species. Many researchers are thus actively investigating the composition of the vesicles, looking in particular for molecular markers that should differentiate classes of EVs, also allowing identification of the cell types from which EVs originate. To identify the EV producers would have important outcomes, especially when we consider that many pathological conditions (cancer in particular, but also neurodegeneration) associate with a higher production of circulating vesicles and their associated contents, which might thus constitute a new kind of biomarker [44,45,46,47,48,49], a sort of “liquid biopsy” [50]. However, these analyses are difficult, also because it has been found that the same cell type can produce vesicles with different contents depending on the different signals received from the environment; for example, it has been demonstrated that colon cancer cells secrete two exosome populations, with different protein contents, from the basolateral, and the apical side, respectively [5,51], and this finding might be valid for all cells with polarity.

Exocarta 2012 [52], a database of molecules found in exosomes, contained information on 11,261 proteins, 2375 mRNAs, and 764 miRNAs, while the last version of the same database, released in 2015 [53], included 41,860 proteins, more than 7540 RNAs, and 1116 lipid molecules.

The fast-increasing number of cited molecules clearly shows that the scientific community is making an effort to develop a deeper knowledge of the structure, role, and mechanism of production of EVs. In general terms, the notion that the EV content is specific is now generally accepted, implying a specialized function for them, and the probable existence of tissue- and cell-specific mechanisms controlling their production (see below).

A further debated point concerns the possible mechanisms through which EVs, produced by a given cell, communicate with surrounding cells. These mechanisms are probably also diverse. Proteins on the vesicle membrane can interact with receptors present on the target cell, and activate them; alternatively, vesicle membrane proteins could be cut off by proteases and the resulting fragments may act as ligands for cell surface receptors on the target cell. Vesicles may also merge with their targets, with direct transfer of their contents into these cells, or they may be internalized by phagocytosis [54].

In addition, it is also to be remembered that part of the vesicles probably breaks into the extracellular environment, releasing various factors that might themselves act as ligands for nearby cells or alternatively modify the extracellular matrix, thus allowing modification of the adhesion and migration properties of the surrounding cells [55,56,57].

In any case, it is clear that EVs can have deep effects on recipient cells, especially in pathological conditions [58,59,60,61,62,63,64]. Moreover, these cargoes can be at least partially transferred into the recipient cells, and there are examples (see below) of all the different categories of molecules that EVs carry with them.

Given the importance of establishing the fate of EVs once released from producing cells, many approaches have been developed for tracking and imaging them, and trying to follow their distribution, targeting, and kinetics in vivo [65].

### 3.1. Proteins

As mentioned above, a variety of proteins with different functions have been identified in vesicles, especially through proteomic analysis [63,64,66]. Moreover, EV-associated proteins have been shown to be transferred to recipient cells: for example, EV-mediated transfer of proteins from oligodendroglioma cells to astrocytes in culture has been clearly demonstrated [67]; similarly, radioactive proteins produced by astrocytes and neurons were shown to be delivered to endothelial cells [39].

In general, EVs must contain different classes of molecules to be formed, to be secreted, and to reach their targets, so, among the many proteins enriched in them, it is possible to find chaperones [67] and proteins involved in membrane targeting, fusion, and trafficking, as well as proteins that participate in the formation of MVB. For example, it has been demonstrated that RAB27A is required for EV secretion in HeLa cervical carcinoma cells [68], and RAB35 in Oli–Neu oligodendroglial precursor cell lines [69]. Moreover, proteins often found in EVs include cytoskeletal components and signal transducers, as well as cytoplasmic enzymes [70,71].

Given this ability to package multiple classes of molecules, inside their lumen or on their membrane, EVs have been hypothesized to represent a “cellular strategy evolved to deliver specific combinations of signals to specific target cells” across cell boundaries [72]. From this point of view, one protein that has recently attracted interest for its involvement in generating epigenetic and functional intratumor heterogeneity is H1.0 linker histone [73]. H1.0, accumulation of which has been traditionally associated with cell terminal differentiation, has been now consistently shown to be downregulated in cancer cells [74]. Moreover, cancer cells can discard H1.0 histone through EVs [75,76]. Interestingly, at least in the case of melanoma cells in culture, cells can also discard the mRNA encoding H1.0 [76].

EVs can also contain growth factors. For example, vesicles purified from primary cultures of rat cortical neurons and astrocytes contain both vascular endothelial growth factor (VEGF) and fibroblast growth factor (FGF)-2, which could promote angiogenesis by stimulating brain capillary endothelial cells, also inducing and modulating formation/maintenance of the blood-brain barrier [77,78]. Similarly, oligodendroglioma cells secrete through EVs pro-apoptotic proteins [67], and metalloproteases able to digest the extracellular matrix [55]. A continuous cross-talk among brain cells seems to exist, based at least in part on extracellular vesicles [39,79,80]. Interestingly, it has been proposed that exosomes can also be transferred among different brain cells pathological proteins, such as: (i) the prion protein (PrP), the infectious particle responsible for various transmissible spongiform encephalopathies [81], (ii) misfolded α-synuclein, involved in Parkinson’s Disease [82,83], and (iii) the amyloid β (Aβ) [84], and hyperphosphorylated tau protein [85], both involved in Alzheimer’s disease.

These observations suggest that pathogens can spread throughout the brain via EVs [15,86]; on the other hand, it has been suggested that the most probable explanation for inclusion of these proteins into EVs is actually a defense mechanism, aimed at eliminating dangerous species from the cell [87].

### 3.2. Lipids

Studies on the lipid content of EVs are still much scarcer than those on proteins and nucleic acids. Many authors evidenced a remarkable enrichment in glycosphingolipids, sphingomyelin, cholesterol, phosphatidylserine (PS), and the monosialoganglioside GM3, both in exosomes and in MVs [88,89,90,91,92]. In particular, MV formation seems to occur preferentially in lipid-rich microdomains of the plasma membrane, such as lipid rafts and caveolae [93,94].

The cell plasma membrane has a known asymmetric structure, in which the two sheets of the membrane have different lipid composition, with, for example, PS mostly found in the inner leaflet. The same asymmetry is found in EV membranes. However, the presence of PS also in the outer leaflet of EVs has sometimes been reported, on the basis of reaction with Annexin V [95]. Actually, the presence of PS in the outer leaflet of cells is normally part of an ‘eat-me’ signal, found on apoptotic cells and apoptotic bodies, as well as on activated blood cells, allowing recognition by macrophages and removal from circulation [96,97]. Thus, the meaning of its presence on EVs is controversial, also because PS might mediate EV removal from circulation before their interaction with the surrounding cells. One possible explanation of external PS finding might be that, because of its membrane curvature, EVs contain a high proportion of its phospholipids on the outer leaflet, with an only apparent external enrichment of PS [95]. However, it has been suggested that PS transfer to the outside might occur only after a while from EV secretion, and that it could really represent a signal to allow elimination of the particles [98]. Finally, it has been reported that EVs produced from human bone marrow mesenchymal stem cells expose PS in the outer leaflet only when produced under hypoxia [99].

Interestingly, enrichment in phospholipids bearing two saturated fatty acyl groups in exosomes, compared to the parent cell membranes, has been noticed, although many monounsaturated acyl groups are also present [92].

It has also been proposed that lipid transport via EVs can be considered a mechanism to transport fatty acids, in addition to serum albumin, lipoproteins, or fatty acid-binding proteins [95]. Moreover, we can suppose that the lipid component is important for sorting proteins and nucleic acids to vesicles/exosomes, by direct interactions.

It is noteworthy that EVs carry bioactive lipids, such as different classes of eicosanoids, as well as intermediates and enzymes required for their synthesis [100]; the presence of these molecules in EVs might have the dual effect of protecting them from degradation while allowing a regulated synthesis of eicosanoids after secretion [101]. Bioactive lipid mediators (such as eicosanoids and free fatty acids) could interact with either G-protein coupled receptors outside the cell, or with nuclear receptors present inside the target cells [102].

### 3.3. Nucleic Acids

As mentioned, vesicles contain different types of ncRNAs, such as miRNAs [5,52], lncRNAs [6], and circRNAs [7]. Moreover, specific mRNAs can be delivered to recipient cells [103], and at least sometimes translated in this new location [8,43], generating a functional protein [9] (see Section 5). As we will discuss below, an important point should be to understand whether the loading of RNAs into vesicles is a random mechanism. In the case of miRNAs, Alix and Argonaute-2 (Ago2) proteins seem to be involved [104]. For mRNAs, it is possible to imagine an important role for RBPs. Several classes of RBPs have indeed been found in exosomes [76,105]. At the same time, some mRNAs are enriched in EVs; for example, FGF7 mRNA is preferentially loaded into mesenchymal stem cells (MSC) EVs, while a random sample of cellular mRNAs is not [106].

EVs do not only contain RNA, but also DNA in different forms (double-stranded as well as single-stranded), and of different origins (mitochondrial DNA, genomic DNA, and retrotransposon elements) [107]. The mechanism allowing DNA sorting into EVs is still unknown, but it has been reported that some DNA molecules travel inside the vesicles (and thus are not accessible to DNase digestion), while some others bind to the outside of the membranes, perhaps in an unspecific manner. Kawamura and colleagues [107] have also shown that EVs obtained from tumor cells and treated with DNase still contain the mutated *KRAS* oncogene. This finding suggests an EV-mediated horizontal transfer of genetic material from cancer cells to their neighbors, although it has not yet been demonstrated whether such DNA is functional in the recipient cells, as the cells contain defense mechanisms against the exogenous DNA integration. For example, Lee et al. [108] demonstrated that EVs carrying an oncogenic *HRAS* DNA failed to permanently transform immortalized fibroblasts.

Extracellular vesicles also contain retrotransposon elements, as well as Long Interspersed Element-1 (LINE-1, L1), ALU repeated sequences, and human endogenous retroviruses (HERV) [109]. Thus, the existence of an EV-mediated mechanism for spreading of these elements towards even remote sites can be hypothesized. Moreover, movable elements might have been exchanged continuously over time via EVs, thus contributing to the evolution of new species.

## 4. Sorting of RNA to Vesicles

Culture media and body fluids usually contain secreted RNA, enclosed in EVs or in free RNP complexes [110]. It has been found that EV RNA content is different in EVs produced by different cell types, and that it changes according to the cell physiological conditions, as well as the EV subcellular source. Among the conditions that strongly influence EV-RNA profiles, of particular importance are hypoxia [111], oxidative stress [112], tumorigenesis [113,114], and infections by different microorganisms, which are supposed to modulate the host’s immune system via EVs [3]. Squadrito and colleagues [115] reported that macrophage activation by interleukin 4 (IL-4) induces significant changes in transcription of specific mRNAs, and of the miRNAs that target them. Variations in the number of specific miRNAs, or their targeted transcripts, may in turn change miRNAs’ localization, moving them from sites of activity (P bodies) to sites of exosome biogenesis (MVB), or vice versa, consequently altering miRNAs’ sorting to exosomes [115]. Many different studies support the idea that EVs are filled with specific RNAs, such as some ncRNAs [116,117] and specific miRNAs [8,9]. Since diverse RNA modifications direct RNA metabolism [118], it is possible to hypothesize that the same modifications, or at least some of them, could also affect the packaging of RNAs into EVs [3]. In one study concerning EVs isolated from human B-lymphocytes, 3′-end-uridylated miRNAs were more abundant in exosomes, while 3′-end-adenylated miRNAs were more abundant in cells [119]. These results suggest that post-transcriptional modifications could contribute to RNA sorting to EVs.

The main problem with RNA sorting is understanding the mechanisms regulating it. Subcellular distribution of RNAs, i.e., localization to specific compartments, might influence the inclusion in EVs and, depending on localization, in different types of EVs. In other words, the regulation of localization of RNAs in the cell, and of their subsequent sorting could share some mechanisms, and maybe also some components. It has been demonstrated that exosome biogenesis relies on the endosomal sorting complexes required for transport (ESCRT), essential for many cell membrane-involving processes, such as plasma membrane abscission, viral budding, and MVB formation. ESCRT has been reported to have a role in sorting mRNA and miRNA cargoes to exosomes [120]. The ESCRT complex comprehends four main (ESCRT 0, I, II, and III), plus some accessory proteins [121]; ESCRT III, in particular, seems to form helical polymers that drive the curvature of the membrane necessary to form exosomes inside the MVB [122,123]. In addition, modification in the relative activity of membrane enzymes responsible for transfer of lipids from a leaflet to the other [124], or for metabolism of membrane lipids (for example, acid sphingomyelinase) can modify the tendency of the membrane to form vesicles [42,125].

Castello et al. [20] recently developed an RNA-binding domains (RBD) map thanks to which they identified, on a proteome-wide scale, 1174 RNA-binding sites within 529 HeLa cell RBPs; their work has allowed for discovering numerous novel RBDs [20]. Interestingly, many of these domains were present in metabolic enzymes. In addition, potential RNA-binding motifs have been also predicted in membrane proteins involved in cell-to-cell and/or cell-to-environment communication, such as connexins [126].

Based on all these findings, one may imagine that inclusion of RNAs in vesicles would reflect their concentration in the vicinity of the sites of EV formation, sites at which RNAs could accumulate because of their interaction with RNA-binding domains present in membrane proteins, or even because of their direct or indirect interactions with the membrane lipids themselves.

In any case, RNAs included in EVs most probably contain specific sequences, and it has been shown that repeated sequences, among which LINE-1 and long terminal repeat sequences (LTRs), are highly represented. An enrichment has been also observed for sequences mapping to transfer RNA (tRNA) loci, or matching Signal Recognition Particle (SRP)-RNA and vault- and Y-RNAs [116,127,128]. Actually, exosomal mRNAs share sequences that could be involved in their sorting and, interestingly, the presence of these sequences correlates with lower stability [129].

In T-cells, a group of miRNAs, found to be enriched in exosomes, shares a specific sequence named the EXOmotif (GGAG), which binds hnRNPA2B1 ribonucleoprotein, probably responsible for miRNA sorting. Most important, sumoylation seems necessary for hnRNPA2B1 function, even though it is not clear whether the modification influences hnRNPA2B1 localization or binding activity [130,131].

Sequences involved in sorting are often present in the 3′-UTR of the mRNAs: one example is mRNA encoding galanin receptor 3 (GalR3), found in human primary glioblastoma multiforme cells and enriched in the exosomes produced by these cells. A CUGCC motif forms a target sequence for miR-1289 and seems, at the same time, to be responsible for GalR3 mRNA sorting [132]. It is thus likely that only RNAs endowed with specific signals are loaded into EVs by an active process. Specific RNA-binding proteins, which also interact with MVB and/or membrane microdomains where the exosomes form, could then direct RNA sorting [133].

One RBP secreted in vesicles, most likely through the endolysosomal compartment, is Y-box protein (YB)-1, a factor involved in proliferation and in cell migration [134], and probably also in RNA secretion. In one study, a set of miRNAs was found to be highly enriched in exosomes selected for the presence of the CD-63 membrane marker, and the authors demonstrated that YB-1 is necessary at least for miR-223 sorting [135]. Other reports indicate that YB-1 binds also to tRNA fragments and small non coding RNAs (sncRNAs) [136,137]. In human embryonic kidney 293 (HEK293) cells, YB-1 interacts with three motifs (ACCAGCCU, CAGUGAGC, and UAAUCCCA) that are enriched in exosomal mRNAs and lcnRNAs. In the same work, it was also shown that the methyltransferase NSUN2 recognizes one of these motifs, CAGUGAGC, and the authors suggested that both YB-1 and NSUN2, by binding to these sequences, could regulate mRNA sorting [138].

Other proteins involved in sorting to exosomes are the endonucleases of the Ago2 family. These proteins control the secretion of some miRNAs, and their activity is inhibited by phosphorylation. In turn, phosphorylation of Ago2 depends on the activation of KRAS and on downstream signaling by MEK-ERK kinase [139]. The role of KRAS on miRNA specific secretion via EVs has also been confirmed in cancer cells [140].

Sorting of miRNAs, and in particular of miR-122, may also involve HuR, which binds RNA by its RRMIII domain. HuR function seems to be regulated by ubiquitination [141]. Santangelo and colleagues [142] noticed selective sorting of specific RNAs (called hEXO-miRNAs) to exosomes of hepatocytes, and identified some components of the sorting pathway, among which Synaptotagmin Binding Cytoplasmic RNA Interacting Protein (SYNCRIP), also known as hnRNP-Q; in particular, SYNCRIP binds to the hEXO motif, present in both miR-3470a and miR-194-2-3p [142].

By a combination of silencing and immunoprecipitation, it has been also demonstrated a role of Annexin A2 in selecting RNA for sorting in EVs, even though it is not clear whether this protein interacts directly with miRNA molecules or indirectly, through other RBPs [143].

## 5. Role of Transferred RNAs in Modifying the Phenotype of Receiving Cells

As discussed, cells can sort to vesicles different kinds of RNA, mostly bound to proteins. However, what is the fate of these RNAs when they reach the extracellular environment? Are they active in the recipient cells, or are EVs simply a way to eliminate waste RNAs? Here we will try to give at least partial answers to these open questions.

### 5.1. Small Non-Coding RNAs

EVs transfer different kinds of ncRNAs [144]. The most studied are miRNAs, small RNAs, about 22 nucleotides in length, able to pair with complementary sequences, present on target RNA transcripts, called microRNA recognition elements (MRE) [145]. Pairing usually results in the repression of target mRNA translation. In 2010, by using a quantitative reverse trancriptase-polymerase chain reaction RT-PCR approach, Pegtel et al. [146] demonstrated that miRNAs encoded by the Epstein Barr Virus (EBV) were secreted by infected B-cells via EVs and internalized by co-cultured Monocyte-Derived Dendritic Cells (MoDC); most important, these EBV-encoded miRNAs could repress in a dose-dependent manner their target genes (such as that for the CXC motif chemokine ligand 11: CXCL11/ITAC) in MoDC [146]. Similarly, secreted monocytic miR-150 was found to enhance migration activity of the recipient endothelial cells [147]. In the same year, Kosaka et al. [148] studied the ability of an exosome-transferred miRNA known to be downregulated in prostate cancer (miR-146a) to restore conditions of growth inhibition in recipient cells; they found that miR-146a was not only able to inhibit cell growth, but did so by knocking down the Rho-associated protein kinase 1 (ROCK1), a known target gene for miR-146a [148].

More recently, cancer-associated adipocytes (CAAs) and fibroblasts (CAFs) were found to secrete via exosomes very high amounts of miR-21 and its iso-miRNAs (variants of the main species). By functional studies, the authors demonstrated that miR-21 is transferred from CAAs or CAFs to ovarian cancer cells, where it suppresses apoptosis while conferring chemoresistance. The authors also showed that miRNA-21 acts by targeting the expression of the gene encoding apoptotic peptidase activating factor 1 (APAF1) [10]. Very similar results have been found in a study on invasive prostate cancer cell lines: cancer cells could activate stromal fibroblasts by secreting EVs, but were also able to promote EV release from activated fibroblasts; in turn, these latter EVs could enhance cancer cell migration and invasion [149]. This kind of observation suggests that a continuous cross-talk exists among cancer cells and their microenvironment, and that it is based at least in part on EV exchange [150,151,152,153].

In spite of these and other examples suggesting that miRNAs can be secreted via EVs and then captured by other cells in which they target specific mRNAs, definitive acceptance of this process is still lacking; first of all, it is possible that miRNAs are secreted and reach surrounding cells through other carriers, such as lipoproteins [154]. Second, to act on their targets, miRNAs need to be part of a silencing complex (the RISC complex), which also contains Ago2. Hence, it is unclear how they manage to form specific complexes once they have arrived in the recipient cell [155]. As mentioned above, Ago2 controls the secretion of some miRNAs, and its regulated sorting to EVs has been reported [139,156]. Thus, it is possible that at least some miRNAs reach recipient cells through EVs in which they are already part of an active silencing complex. A further problem concerns the real impact of EVs on recipient cells. Chevillet et al. [157] have shown, for example, that most exosomes derived from standard preparations do not harbor many copies of miRNA molecules, thus suggesting that a stoichiometric analysis should be of the utmost importance in evaluating the possible function of EV populations [157].

Actually, miRNAs are not the only small RNAs in EVs. By high-throughput small RNA sequencing, a highly abundant small RNA derived from the 28S ribosomal (rRNA) has been identified [158], and, as mentioned above, specific fragments of Y-RNAs and tRNAs [127] are enriched in EVs. The real function of these molecules is still under study; it has been recently reported, for example, that a Y-RNA fragment (EV-YF1) is the most abundant small RNA in EVs released by cardiosphere-derived cells and that these YF1-containing EVs confer cardioprotection after ischemia/reperfusion of the heart [128]. However, the mechanisms that allow these RNA fragments to affect the recipient cells are still unclear.

### 5.2. Long Non-Coding RNAs

Besides small RNAs, EVs also contain lncRNAs [6]. lncRNAs have now been recognized to represent an important class of transcripts in all organisms, with increasing impact along the evolutionary ladder. They are involved in a variety of regulatory functions, such as X chromosome inactivation, imprinting, control of chromatin structure, and control of mRNA metabolism [159,160,161,162]. Interestingly, the genomic regions from which they are transcribed have chromatin organization indistinguishable from mRNA-transcribing genes, in that they have the same histone modifications; moreover, they are transcribed by RNA polymerase II and, in most cases, the transcripts are spliced, capped, and polyadenylated [13]. Probably the most interesting among the known functions of lncRNAs is their epigenetic role: some of them guide chromatin-modifying enzymes to specific genome sites and/or act as a scaffold for chromosomal organizing factors [12] and transcription factors [163]. Given their roles, it is not surprising that their expression is regulated in development and differentiation [164,165], and that their abundance can change in pathological conditions [166]. Intriguingly, it seems that the human cerebral cortex uses for its development and functions the highest amount of ncRNA with respect to any other organ in the body; the non-coding part of the genome probably gives our brain its astonishing complexity, and, at the same time, higher vulnerability, because of the higher number of regulatory genomic regions that can be altered, resulting in brain malfunctioning [167,168]. A further role suggested for lncRNAs is to function like competing endogenous RNA (ceRNA) to regulate miRNA action: in other words, they can contain the same MREs as mRNA targets, and can actively compete with them, thus buffering miRNA function; because of this role, they were also named ‘miRNA sponges’ [169]. It has been suggested that the sponge function also depends on the localization of the lncRNA [170].

Now, lncRNAs are enriched in EVs, and specifically sorted to them [6]. Ahadi et al. [171] have found, for example, that specific lncRNAs, which harbor miRNA seed regions, are enriched in exosomes released from four prostate cancer cell lines; these authors also reported that these lncRNAs contain RBP-recognition motifs [171]. Inclusion of lncRNAs into EVs, and probably their specific sorting to them, might have two consequences: (i) to discard competitors, thus modifying the percentage of the corresponding active miRNAs, and (ii) to transfer the competitors into other cells in which active miRNA concentration should be now altered. As in the case of miRNAs, although their presence in extracellular vesicles suggests a function in the recipient cells, it is still debated whether lncRNAs carried by EVs actually have a function once received, and, if so, through which mechanisms. Evidence is available, however, on the effects of EV-transferred lncRNAs. Takahashi et al. [172] have reported, for example, that hepatocellular carcinoma (HCC) produces EVs and that these EVs could contain and transfer lncRNAs, among which is linc-VLDLR (very low density lipoprotein receptor); on the basis of their data, the authors speculate that linc-VLDLR, once received, is responsible for the reduced chemotherapy-induced cell death of recipient cells [172].

By labeling RNAs produced by HeLa cells in culture and exposing C33A cells to EVs produced by labeled HeLa cells, Hewson et al. [173] found that a specific class of EV-associated lncRNAs (denominated Exo 1–4) was transferred from producing cells to recipient ones. On the basis of their results, the authors also suggested that these lncRNAs were responsible for the cell viability enhancing effects of HeLa-released EVs [173]. By immunoprecipitation of RNA–protein complexes formed after transfecting the cells with biotin-labeled lncRNAs, the authors also found that Exo1–4 lncRNAs directly interacted with lactate dehydrogenase B (LDHB), high-mobility group protein 17 (HMG-17), and colony stimulating factor 2 receptor beta subunit (CSF2RB), proteins involved in metabolism, nucleosomal architecture, and cell signalling, respectively [173].

### 5.3. mRNAs

In 2007 it was shown that, after treating human cells in culture with mouse exosomes, mouse proteins could be found in the recipient cells, indicating that mouse mRNAs, transferred by EVs, could be translated [8]. The next year, Skog et al. [9] demonstrated by RT-PCR that EVs purified from glioblastoma cells, transduced with a lentiviral vector encoding a secreted luciferase from Gaussia (Gluc), secreted the corresponding mRNA into EVs; moreover, microvesicle-delivered mRNA was translated by recipient cells to which the purified EVs had been added [9]. Similar observations were also done through in vivo experiments; the transfer of functional Cre recombinase mRNA from immune cells to neurons, via purified EVs, was clearly demonstrated after intracerebellar injection of Cre mRNA-containing EVs [174,175]. Similarly, Lai et al. [176], by using a double labeling strategy, were able to visualize the kinetics of EV production, release, and transfer, as well as of their mRNA cargo, further revealing the temporal dynamics of EV uptake and translation of EV-delivered mRNAs [176]. More recently, it was demonstrated that kidney tubular cells, which do not normally express interleukine 10 (IL-10), become able to do it after exposure to MSC-derived EVs containing IL-10 mRNA [105].

All these observations suggest that, at least in some cases, messages enriched in EVs can be actively translated in recipient cells, thus ensuring the horizontal transfer of properties from one cell to another. Based on what we have learned about the different classes of ncRNAs, it is also possible to hypothesize that the specific combination of mRNAs and ncRNAs present in EVs has an additive effect on the overall response of the recipient cells. Moreover, it has to be considered that coding mRNAs can also be involved in a number of non-coding functions. Thus, it is not sufficient to know that a given mRNA is transferred from one cell to another if we do not know which other species of interacting RNAs and proteins are also included into EVs.

### 5.4. Circular RNAs

In 1976, it was reported that viroids, uncoated infectious RNAs that are pathogenic for certain plants, are covalently closed circular RNA molecules [177]. In the same year, it was also found that RNA isolated from nucleocapsids of cells infected with different strains of Sendai virus mainly consisted of defective interfering (DI)-RNAs, the main property of which was their ability to form circular structures [178]. Interestingly, a few years later, some mitochondrial RNAs were also found to be covalently closed to form circles [179]. For many years, however, circular RNA molecules identified in different cell types were considered junk RNA derived from primary transcript splicing [179,180,181,182]. In the last 4–5 years, evidence has been accumulating that circRNAs not only exist, probably in all organisms, but constitute a stable and conserved form of RNA, which might fulfill a lot of different and regulatory functions [183,184]. It has been suggested that they function as miRNA sponges, able to bind and trap specific miRNAs [11,185]. For example, a circRNA identified in the human and mouse brain has been found to act as a sponge for miR-7 and consequently was termed ciRS-7 (circular RNA sponge for miR-7) [186]. The same circRNA acts as an oncogene in HCC, by targeting miR-7 [187].

circRNAs are mainly produced by a particular type of alternative splicing called ‘back-splicing’ [188]; one of their most important properties is their exceptional stability and long half-lives [189], probably due to the fact that 3′- and 5′-ends are closed together and therefore not accessible to exonucleolytic degradation.

circRNAs have been classified in different ways, the most useful probably being their subdivision into exonic, intronic, antisense, sense overlapping, and intergenic circRNAs, depending on their genomic origin [190]. The existence of different kinds of circRNA corresponding to different classes of DNA sequences suggests that they might also have differentiated functions in cells. Moreover, they have been reported to be tissue- and developmental stage-specific [191].

Interestingly, a correlation has been suggested between the global abundance of circRNAs and the rate of proliferation of tissues, with a higher amount of circRNAs in low-proliferating cells. As a matter of fact, circRNAs are highly represented in the brain [192], while, for example, in colon cancer tissue circRNAs are less concentrated in cancer cells than in the surrounding non-cancerous tissue. To explain this finding, it has been proposed that the population of circRNAs might be divided at mitosis among the daughter cells, thus reducing the total number in rapidly dividing cells [193]. In contrast, however, it has been reported that circ-TTBK2 RNA is upregulated in glioma tissues and cell lines, while linear TTBK2 RNA is not dysregulated. TTBK2 RNA encodes Tau Tubulin kinase 2 and enhanced expression of its circular form promotes cell proliferation, migration and invasion, while inhibiting apoptosis; the mechanism underlying this effect involves a sponge action of circ-TTBK2 on the onco-suppressor miR-217, which in turn targets the mRNA encoding oncogenic HNF1beta [194].

Interestingly, some circRNAs do not have any miRNA binding sequence, and regulate gene expression by binding and modulating RNA polymerase II [195], perhaps cooperating with RBPs involved in mRNA metabolism.

On the other hand, RBPs themselves can be involved in circRNA formation. For example, in epithelial cells undergoing epithelial–mesenchymal transition, Quaking RBP is activated in response to TGF-β and acts, in turn, as a stimulator of circRNA biogenesis [23].

It is also important to remember that circRNAs might in principle be closed mRNAs, which could be translated if they contain an internal ribosome entry site (IRES) [196], even when they do not have a classical one [190,197]. Recently, a database (circRNADb) has been created that contains 32,914 human exonic circRNAs [198,199], but, up to now, translation has been clearly demonstrated only for the hepatitis D virus (HDV) [200].

Coming back to EVs, circRNAs, which are normally cytosolic molecules, have been found in extracellular vesicles, even enriched with respect to the producing cells [201], as well as with respect to the corresponding linear RNAs [7,202]. In their study, Lasda and Parker [202] used three different approaches to validate the conclusion that circRNA was really associated with EVs: electron microscopy of each EV preparation, nanoparticle tracking analysis, and mass spectrometry to confirm composition of each EV preparation.

Given the already mentioned cell specificity of circRNAs, and the observation that several circRNAs are modified in tumorigenesis, the stable, EV-associated RNA molecules in the blood of patients with different kind of cancers are now considered as promising biomarkers [190,199,203,204,205]. However, it is unclear why circRNAs are enclosed in EVs and what the fate and the activity, if any, of circRNAs after delivering to recipient cells might be. Given the nuclease resistance of circRNAs, one hypothesis is that cells, unable to degrade them, use EVs just to discard them [202]. It is, however, also possible that circRNAs are released only when in excess; in this case, circRNAs might be captured by recipient cells and alter their behavior. As in the case of the other RNA species, we do not yet have clear evidence that this actually happens. Since, however, it has been demonstrated that miRNAs transferred by EVs can efficiently repress mRNA translation in recipient cells, and given the sponge activity of circRNAs, we can suppose that EV-associated circRNAs can have an activity both in the EVs themselves (as miRNA and perhaps protein transporters), as well as in the recipient cells.

## 6. RNA as a Protein Carrier

In Section 5, we discussed the importance of the different species of RNA in transforming the recipient cells once transferred via EVs. We have also discussed the importance of RNA-binding proteins in cellular RNA metabolism and localization (Section 2), further including in the latter function the possible role of RBPs in localizing RNAs to sites from which they are then sorted to EVs (Section 4). Up to now, in other words, we considered the proteins as involved in the formation of RNA–protein complexes, fundamental for sorting and transferring RNA, and, on the other hand, the different species of RNAs as the real protagonists, each with its peculiar role, of recipient cell phenotypic modification.

In this section, we will propose a different point of view, in which RNA behaves as a carrier, and the transferred proteins are the real protagonists of the epigenetic effects on recipient cells.

Our hypothesis starts from the observation that many RBPs can bind both RNA and DNA. Among these proteins, just to give a few examples, we can consider: (i) the histones themselves; for example, a role in RNA processing has been recently suggested for the mouse ortholog of human H2A.B histone [206]; (ii) the cold-shock domain-containing proteins, such as CSD-C2 (also known as PIPPin) [207], and YB-1, which is both a transcription factor and a component of mRNPs [208]; (iii) proteins containing zinc finger domains: these proteins were initially considered transcriptional regulators but can also bind RNA [209]. Besides these proteins that contain well known RNA/DNA binding domains, proteins lacking conventional nucleic acid-binding motifs have been also found; recently it has been reported, for example, that SOD 1, the metalloenzyme known for catalytically eliminating superoxide radicals, also plays important roles as a transcription factor and an RBP [30].

The presence in EVs of a number of transcriptional regulators has been reported by Ung et al. [210], based on exosome proteomics; among the proteins identified, some (i.e., BRCA1-Associated RING Domain Protein 1, BARD1; Far Upstream element Binding Protein 1, FUBP1; Hepatocyte nuclear factor 4-alpha, HNF4A; Replication factor C subunit 1, RFC1; SWI/SNF related, matrix associated, actin dependent regulator of chromatin, subfamily a, member 1, SMARCA1; and Zinc finger and SCAN domain containing 16, ZSCAN16) have a direct and known transcriptional activity, while many other proteins can be better considered multifunctional [210].

Thus, we asked whether some DNA/chromatin-binding proteins present in EVs might also behave as RBPs.

Among the proteins able to bind both DNA and RNA, and certainly present in EVs, one is, as mentioned, YB-1, which is actively secreted through a non-classical pathway, and is protected by microvesicles from protease degradation. In this form, it exerts a potent proliferative effect and promotes cell migration; both actions seem to require its cold shock domain [134]. Another is the myelin expression factor 2 (MYEF-2), first described for its ability to repress transcription of the gene encoding myelin basic protein [211], but recently found in EVs released by melanoma cells, bound to H1.0 histone mRNA [76].

Among the proteins involved in transcription and found in EVs by Ung et al. [210], FUBP1 is also thought to bind RNA, and the same is probably true for many other proteins. This means that at least some EV-transferred proteins have the potential to ride RNA (thus used as a Trojan horse) for arriving to the recipient cells, where they can then contribute to modifying the chromatin organization of genes and, hence, their expression.

Of course, the two possibilities (RNA–protein complexes as RNA carriers or as protein carriers) are not mutually exclusive, and the main problem, still to be solved, is how cells choose what is “to be sent” outside and what is “to be used” out of the received packages.

## 7. Conclusions

EVs represent an astonishing tool to transfer biochemical properties from cell to cell, and perhaps an ancient way to level individual potentials within a population, in spite of genetic variability. Central protagonists of EV-mediated exchanges are RNA–protein complexes, formed by a variety of RNA species and by multifunctional RNA-binding proteins. Proteins and RNAs are fundamental for complex formation and both components have a function in driving themselves out of the producer cells and then inside the recipient ones. However, many points remain to be clarified. First, how are proteins and RNAs taken apart in order to start functioning, once in the recipient cell? RNA–protein complexes travelling inside the cell, for example along an axon or a dendrite in a neuron, are not translationally active during the trip, but they can be activated locally by specific signals. Is the entrance into a new cellular environment such a signal for the complexes present in EVs? May the complexes remain silent even inside the new environment unless further signals are delivered? If the RNA-bound proteins can act like chromatin modifiers and/or transcription factors, when and how may they start doing so? The same questions, of course, pertain to RNAs of all classes.

In conclusion, in recent decades we have learned a lot about extracellular vesicles and their cargoes. We also know that EVs and their contents can probably be used as useful biomarkers in pathologies such as cancer and neurodegeneration. Much work is still necessary, however, in order to understand the biochemical details of EV formation and delivery, as well as of cargo activation, utilization, and, perhaps, destruction.

## Supplementary Materials

Supplementary File 1
