# Supplementary material for: Extracellular Vesicle-Associated RNA as a Carrier of Epigenetic Information
Source: Genes (Basel). 2017 Sep 22;8(10):240. doi: 10.3390/genes8100240 (PMC5664090; doi:10.3390/genes8100240)
Supplement: Supplementary File 1 [file genes-08-00240-s001.pdf]

Legend to graphic abstract

Two cells, embedded in the extracellular matrix (ECM), are shown. For simplicity, EV traffic is shown from cell A (producer cell) to cell B (recipient cell) only. In the organism, however, EV-mediated communication is probably reciprocal.

EVs can originate: i) by direct budding at the plasma membrane (e.g. ectosomes or membrane vesicles), possibly at the level of specific microdomains, such as lipid rafts (LR), or, ii) by exocytosis of multivesicular bodies (MVB) (e.g. exosomes), which are part of the endosomal system.

Three classes of RNA which have been found in EVs are shown: **a)** pre-miRNAs exit the nucleus through the nuclear pores and, once in the cytoplasm, are transformed into mature miRNAs; miRNAs can base pair with their target mRNAs and with RNA-binding proteins (RBPs), among which Ago2, forming ternary miRNA-mRNA-RBP complexes; **b)** during transcription and maturation in the nucleus, mRNA binds to RBPs, forming ribonucleoprotein complexes (mRNPs); some RBP remain bound during transport, and, by attaching directly or indirectly to cytoskeletal elements, control mRNA subcellular localization and, perhaps, its sorting to EVs; **c)** some RNA species (both coding and non-coding) can close, forming circles, which can interact with miRNAs (sponge effect) and with RBPs, forming circRNA-miRNA-RBP complexes. Sorting of all these complexes to EVs might be controlled by interactions of RNA-protein complexes with membrane lipids and/or cell membrane proteins (P_1, P_2, P_3), via RNA-binding domains of membrane proteins, and/or specific RNA nucleotide sequences/RNA modifications/RNA secondary structures.

An enlarged view of an EV and its content is shown in the insert. ER, endoplasmic reticulum; GA, Golgi Apparatus; EE, early endosomes,; LE, late endosomes.

Once released EVs may have different fates: **d)** they might be recognized and bound by specific receptors on the recipient cells; **e)** they can fuse with the recipient cells; **f)** they can break, releasing their content that might be then destroyed in the ECM, and/or reach the recipient cells through interaction with their membrane receptors. Because of EV break in the ECM, or because of other secretion pathways, all the above cited RNA-protein complexes could be EV-free in the ECM ($\mathbf{g_1}$, $\mathbf{g_2}$, $\mathbf{g_3}$).

In any case, both the different species of RNA, and proteins can reach the recipient cells, and, as discussed in the text, modify gene expression. In particular, we discuss the idea that, at least in some case, RNA can function as a carrier of regulatory proteins (pathway "**h**"), directly involved in the epigenetic modification of chromatin structure and function.
